# Correlation of Fractional Excretion of Sodium With Renal Scintigraphy and Renal Ultrasonography in Children With Unilateral Ureteropelvic Junction Obstruction Undergoing Pyeloplasty: A Prospective Observational Study

**DOI:** 10.7759/cureus.113947

**Published:** 2026-08-04

**Authors:** Jagdish B, Enono Yhoshu, Manish K Gupta, Sandeep Saini, Rajat Piplani, Intezar Ahmed, Satya Sree Balija

**Affiliations:** 1 Pediatric Surgery, All India Institute of Medical Sciences, Rishikesh, IND; 2 Nephrology, All India Institute of Medical Sciences, Rishikesh, IND

**Keywords:** children, differential renal function, dtpa, fractional excretion of sodium, pediatric urology, pyeloplasty, radionuclide imaging, ureteropelvic junction obstruction

## Abstract

Introduction

Ureteropelvic junction obstruction (UPJO) remains a common pediatric urological condition that often requires surgical intervention, and the search for reliable markers to assess postoperative resolution is ongoing. Partial ureteral obstruction is associated with impaired sodium reabsorption; consequently, animal studies have consistently demonstrated natriuria in models of partial ureteral obstruction. Fractional excretion of sodium (FENa) reflects tubular function by measuring urinary sodium excretion and has been used to differentiate the types of acute kidney injury (AKI). To date, no published studies have evaluated preoperative and postoperative FENa in patients with UPJO to demonstrate postoperative improvement or to determine its correlation with established modalities for assessing renal function, such as renal scintigraphy.

Aims

This study aimed to evaluate the correlation between FENa and renal scintigraphy, specifically diethylene triamine pentaacetic acid (DTPA) renography, as well as renal ultrasonography (USG), in children with unilateral UPJO before and after pyeloplasty.

Methodology

A time-bound prospective observational study was conducted in the Department of Pediatric Surgery from October 2021 to April 2023. In children who met the diagnostic criteria for UPJO and required surgical intervention, a urine sample was collected intraoperatively to determine the FENa. The FENa values were then correlated with the findings of renal scintigraphy and renal USG.

Results

A total of 35 patients with a mean age of 7.03 ± 5.03 years were included in the study. FENa on the affected side showed a significant inverse correlation with differential renal function (DRF) (r = -0.650, p < 0.001) and renal cortical thickness on USG (r = -0.413, p = 0.009). In contrast, FENa demonstrated a significant positive correlation with the anteroposterior pelvic diameter (APPD) measured on USG (r = 0.506, p = 0.001).

Discussion

Our findings indicate that improvement in DRF was associated with a reduction in the FENa, suggesting improved tubular sodium handling following pyeloplasty. Greater pelvic dilatation was associated with higher FENa values, both of which decreased after pyeloplasty. Similarly, an increase in renal cortical thickness was associated with a reduction in FENa following surgery. These findings suggest that the FENa may serve as a useful surrogate marker for assessing renal function in patients with unilateral UPJO and for evaluating postoperative improvement in renal function following pyeloplasty.

## Introduction

Ureteropelvic junction obstruction (UPJO) is a congenital functional impairment of urine outflow from the renal pelvis to the proximal ureter, which may lead to progressive renal parenchymal damage if left untreated. It is the most common cause of persistent hydronephrosis in the pediatric population [1]. Surgical intervention is indicated in children with UPJO who present with symptoms or evidence of progressive obstruction or declining renal function.

Evidence from fetal studies suggests that impaired renal function is associated with the production of isotonic urine, whereas physiologically normal fetal kidneys produce hypotonic urine because of intact tubular reabsorptive function [2]. Alterations in tubular function secondary to ureteral obstruction have been extensively investigated in animal models. Partial unilateral ureteral obstruction impairs tubular sodium reabsorption, resulting in natriuria [3]. Obstructive uropathy results not only from mechanical impedance to urinary flow but also from secondary alterations in glomerular hemodynamics, tubular transport mechanisms, and interstitial responses, all of which contribute to progressive renal dysfunction [4].

The fractional excretion of sodium (FENa) is calculated using urinary and serum sodium concentrations together with urinary and serum creatinine levels. By reflecting the proportion of filtered sodium that is excreted in the urine, FENa serves as an indirect marker of renal tubular function [5].

Chiou et al. evaluated the FENa in children with UPJO and examined its correlation with postoperative diethylene triamine pentaacetic acid (DTPA) renography findings [4]. However, there is a paucity of literature evaluating both preoperative and postoperative FENa in children with UPJO and correlating these findings with established modalities for assessing renal function and postoperative recovery.

The objective of this study was to evaluate preoperative and postoperative FENa in children with UPJO undergoing pyeloplasty and to investigate its association with changes in differential renal function and ultrasonographic parameters.

## Materials and methods

This prospective observational study was conducted in the Department of Pediatric Surgery at a tertiary care center from October 2021 to April 2023 after obtaining approval from the Institutional Ethics Committee (IEC/21/602). The study included all patients aged 0-18 years with a diagnosis of unilateral UPJO who underwent open, laparoscopic, or robotic pyeloplasty. Patients with a solitary kidney, bilateral UPJO, horseshoe kidney, or other associated urinary tract anomalies were excluded.

Surgical intervention was performed in patients with impaired renal function, defined as DRF ≤40% or a decline of >10% on serial renograms, progressive hydronephrosis with abrupt narrowing at the pelviureteric junction on serial ultrasonography (USG), or symptoms including recurrent flank pain, an abdominal mass, febrile urinary tract infection (UTI), or hematuria.

Methodology

All patients who met the study eligibility criteria and whose parents or legal guardians provided written informed consent were enrolled. Baseline evaluation included kidney function tests (KFTs), kidney-ureter-bladder USG (KUB USG), and renal scintigraphy to assess the severity of hydronephrosis and the degree of renal functional impairment before surgery. On USG, the anteroposterior pelvic diameter (APPD), renal cortical thickness, and grade of hydronephrosis of the affected kidney were recorded. Renal dynamic scintigraphy using technetium-99m diethylene triamine pentaacetic acid (99mTc-DTPA) renography with the F-0 protocol was performed preoperatively to assess renal function. The evaluated parameters included DRF, half-time of tracer clearance (T½), time to peak tracer activity (Tmax), and drainage pattern. T½ was defined as the time required for renal tracer activity to decrease to half of its peak value, whereas Tmax was defined as the interval from tracer injection to the attainment of peak renal activity.

Preoperatively, all patients were kept nil per oral in accordance with the American Society of Anesthesiologists (ASA) fasting guidelines. Standardized perioperative maintenance fluids and anesthetic medications were administered to all patients to minimize variability in hydration status and potential drug-related effects on the FENa. Patients underwent open, laparoscopic, or robotic Anderson-Hynes dismembered pyeloplasty at the discretion of the operating surgeon. In patients with a poorly functioning kidney (baseline DRF ≤ 10%), a percutaneous nephrostomy (PCN) tube was inserted under USG guidance, followed by repeat 99mTc-DTPA renography after a minimum interval of two months to reassess renal function.

Serum sodium and creatinine concentrations were measured as part of the KFT. Intraoperatively, urine samples representative of both the affected and unaffected kidneys were collected for the estimation of urinary sodium and creatinine concentrations. For the affected kidney, approximately 2-3 mL of urine was aspirated from the dilated renal pelvis using a needle before dismemberment of the pelviureteric junction. For the contralateral kidney, urine collected from the urinary bladder through a Foley catheter after dismemberment of the renal pelvis and before pelviureteric anastomosis was used as a surrogate for urine from the unaffected kidney. Although this specimen was not exclusively derived from the contralateral kidney, it was considered representative of the unaffected side. All patients subsequently underwent insertion of a double-J (DJ) stent following completion of the pelviureteric anastomosis.

In patients requiring PCN insertion, the urine sample was collected within one hour of the procedure to accurately reflect the renal functional status at the time of intervention.

FENa was calculated individually for both the renal pelvis, using the formula:

$n=\frac{U.Na\times S.Cr }{U.Cr\times S.Na}\times 100$

where U. Na = urinary sodium, U. Cr = urinary creatinine, S. Na = serum sodium, S. Cr = serum creatinine

The FENa was then compared with the values obtained post-pyeloplasty.

**Figure 1:**
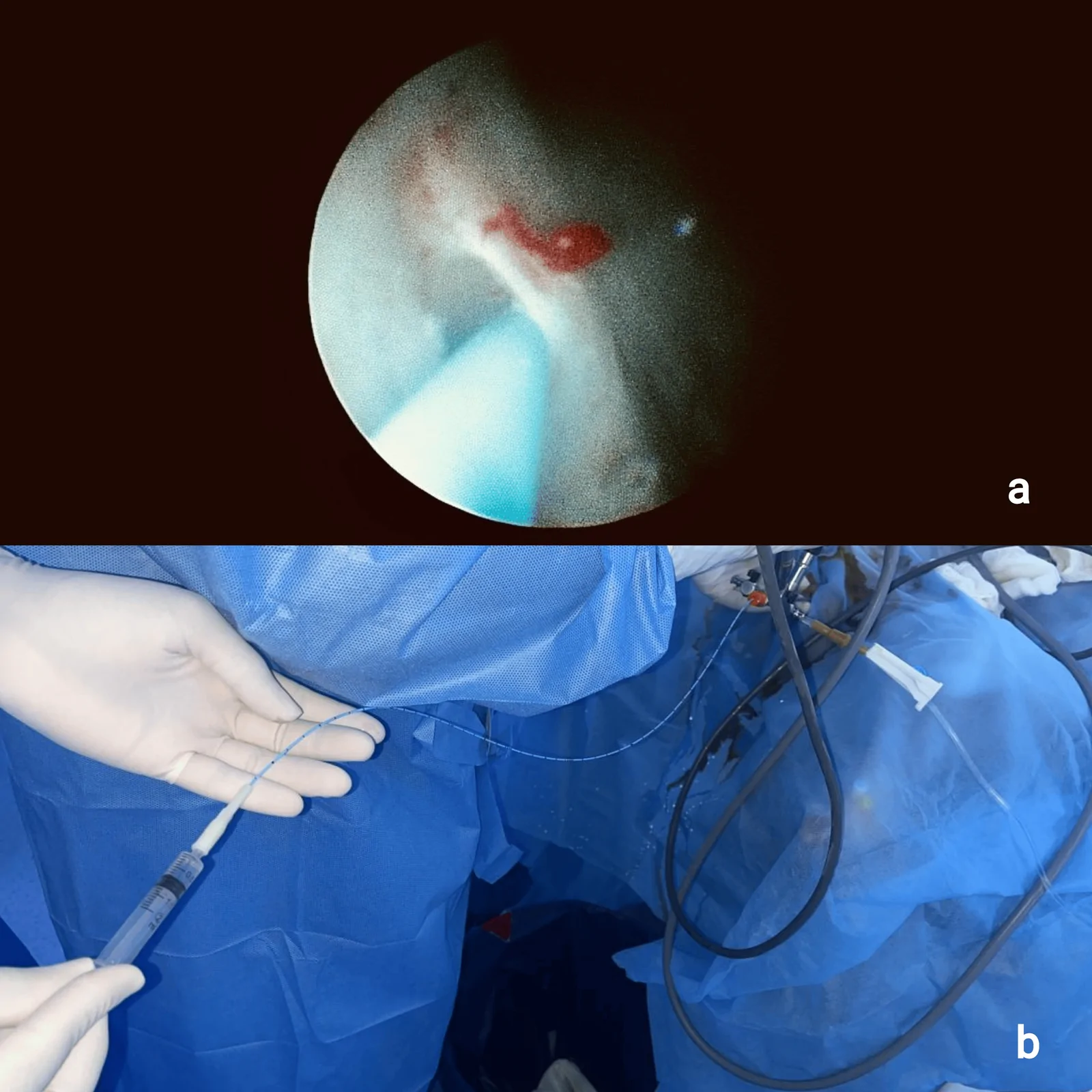
Urine sample collection for postoperative analysis. (a) Cystoscopic view showing a ureteric catheter (blue) inserted into the right ureteric orifice following DJ stent removal. (b) Aspiration of urine with a 2-mL syringe through the ureteric catheter.

At least six weeks after surgery, a repeat KFT was performed, and the DJ stent was removed. During cystoscopy, approximately 2-3 mL of urine was collected separately from each ureter using an age-appropriate ureteric catheter, while ensuring that no trauma was caused to the ureter or bladder (Figure 1).

**Figure 2:**
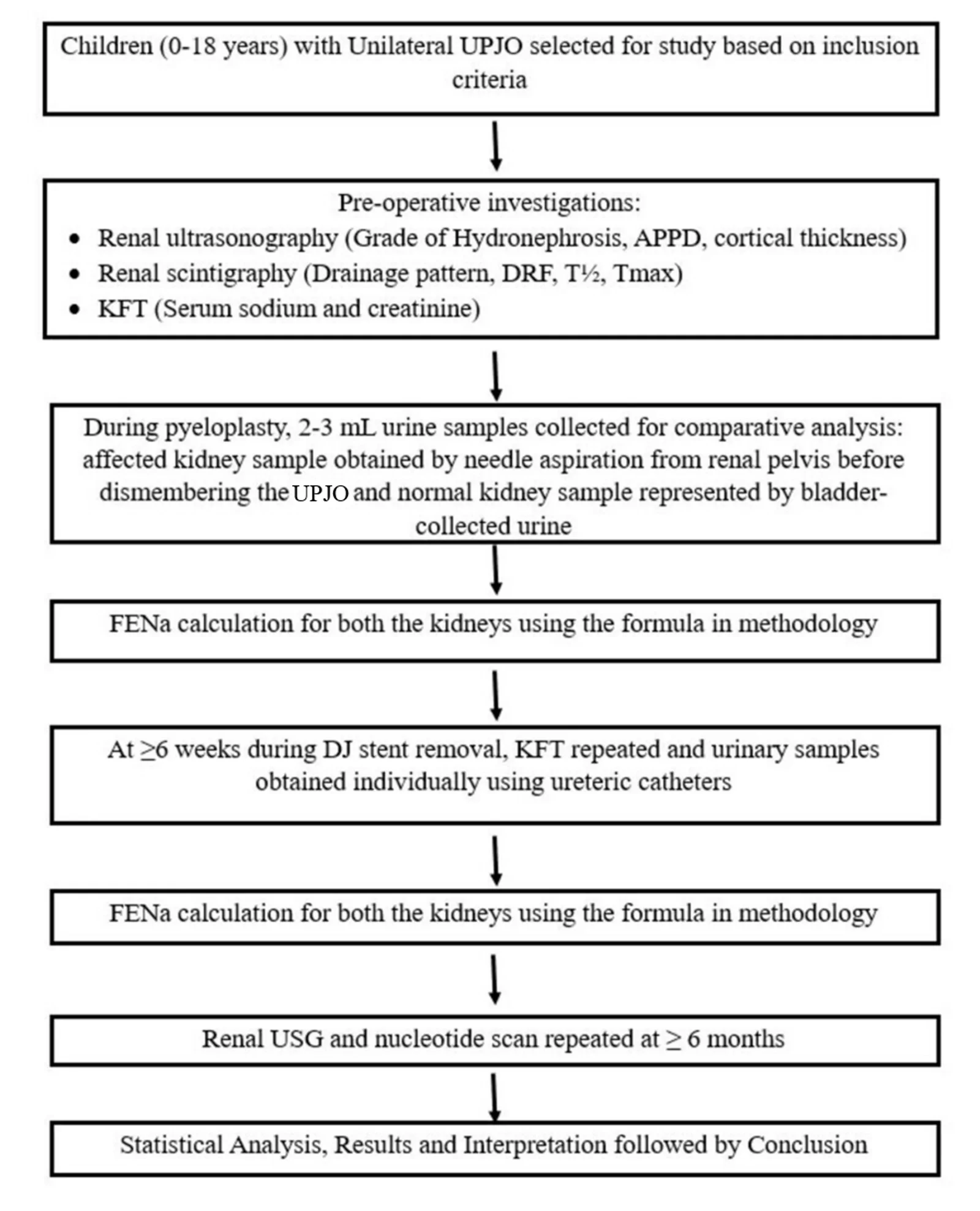
Algorithm depicting the methodology of the study. UPJO: ureteropelvic junction obstruction, APPD: anteroposterior pelvic diameter, KFT: kidney function test, FENa: fractional excretion of sodium, DJ: double-J, USG: ultrasonography.

The FENa was then calculated using the aforementioned formula. All patients underwent follow-up evaluation with renal USG and 99mTc-DTPA renography at least six months after surgery. The study methodology is summarized in the flowchart presented in Figure 2.

Data were coded and entered into a Microsoft Excel spreadsheet (Microsoft Corp., Redmond, WA, USA). Statistical analysis was performed using IBM SPSS Statistics for Windows, Version 23.0 (Released 2015; IBM Corp., Armonk, NY, USA). Comparisons of continuously distributed variables before and after surgery (including FENa, DRF, T½, Tmax, APPD, and renal cortical thickness) were performed using the paired-samples t-test. Pearson's correlation analysis was used to evaluate the relationships between normally distributed continuous variables, including FENa and DRF, FENa and APPD, and FENa and renal cortical thickness. Spearman's rank correlation analysis was used for non-normally distributed or ordinal variables, including FENa and the grade of hydronephrosis, as well as FENa and the drainage pattern. A two-tailed p-value of <0.05 was considered statistically significant.

## Results

A total of 35 patients who met the inclusion criteria during the study period were enrolled. The mean age of the study population was 7.03 ± 5.03 years (range, 6 months to 17 years). The male-to-female ratio was 2.9:1. The right kidney was affected in 18 of 35 patients (51.4%), while the left kidney was affected in 17 of 35 patients (48.6%). The baseline ultrasonographic, renal scintigraphic, and biochemical characteristics of the study population are summarized in Table 1.

**Table 1:** Comparison of preoperative and postoperative parameters of the affected kidney on ultrasonography, renal scintigraphy, and biochemical assessment. A paired sample t-test was done for the above parameters. APPD: anteroposterior pelvic diameter, DRF: differential renal function, DTPA: diethylene triamine pentaacetic acid, FENa: fractional excretion of sodium.

Investigation	Parameters of the affected kidney		Mean	SD	t-value	p-value
Renal ultrasonography	APPD (cm)	Preoperative	3.857	2.520	-6.469	<0.001
		Postoperative	3.449	2.400		
	Renal cortical thickness (mm)	Preoperative	7.857	4.974	7.104	<0.001
		Postoperative	10.53	5.066		
Renal scintigraphy	DRF on DTPA (%)	Preoperative	39.63	8.633	4.163	<0.001
		Postoperative	43.60	5.817		
	T½ in DTPA (minutes)	Preoperative	43.12	11.47	-11.59	<0.001
		Postoperative	17.89	3.930		
Biochemical parameters	Urinary sodium (mmol/L)	Preoperative	87.51	30.19	-2.614	0.013
		Postoperative	79.03	35.66		
	FENa (%)	Preoperative	2.563	2.146	-4.705	<0.001
		Postoperative	1.809	1.977		

Renal USG parameters

The mean APPD was 3.9 ± 2.5 cm (range, 1.1-13.0 cm), and the mean renal cortical thickness was 7.9 ± 5.0 mm (range, 1-18 mm). The APPD decreased significantly following surgery compared with the preoperative measurement (p < 0.001). In contrast, renal cortical thickness increased significantly after surgery (p < 0.001). According to the Society for Fetal Urology (SFU) grading system, all patients had grade 3 or higher hydronephrosis on preoperative assessment, with a significant reduction in hydronephrosis grade following surgery (p = 0.006).

Technetium-99m DTPA (Tc-99m DTPA) parameters

The mean preoperative DRF was 39.6 ± 8.6% (range, 10%-49%), the mean T½ was 43.1 ± 11.5 minutes (range, 14.8-66.5 minutes), and the mean Tmax was 17.2 ± 5.9 minutes (range, 5.9-29.2 minutes). However, T½ and Tmax could not be determined in some patients because the renographic time-activity curve continued to rise throughout the acquisition period.

Postoperatively, DRF improved significantly (p < 0.001), while both T½ and Tmax decreased significantly (p < 0.001). Overall, 27 of 35 patients (77.1%) demonstrated stable renal function (defined as a change in DRF of ≤5%), whereas 8 patients (22.9%) showed improvement (defined as an increase in DRF of >5%).

Preoperatively, the drainage pattern was obstructive in 33 of 35 patients. In the remaining two patients, the drainage pattern could not be assessed because of severely impaired function of the affected kidney. Postoperatively, all patients demonstrated an improvement in the drainage pattern.

Two patients had a baseline DRF of 10% on 99mTc-DTPA renography and subsequently underwent PCN insertion. Repeat renal scintigraphy performed two months later demonstrated improvement in both renal function and drainage in these patients, following which they underwent pyeloplasty.

FENa of the affected kidney

**Figure 3:**
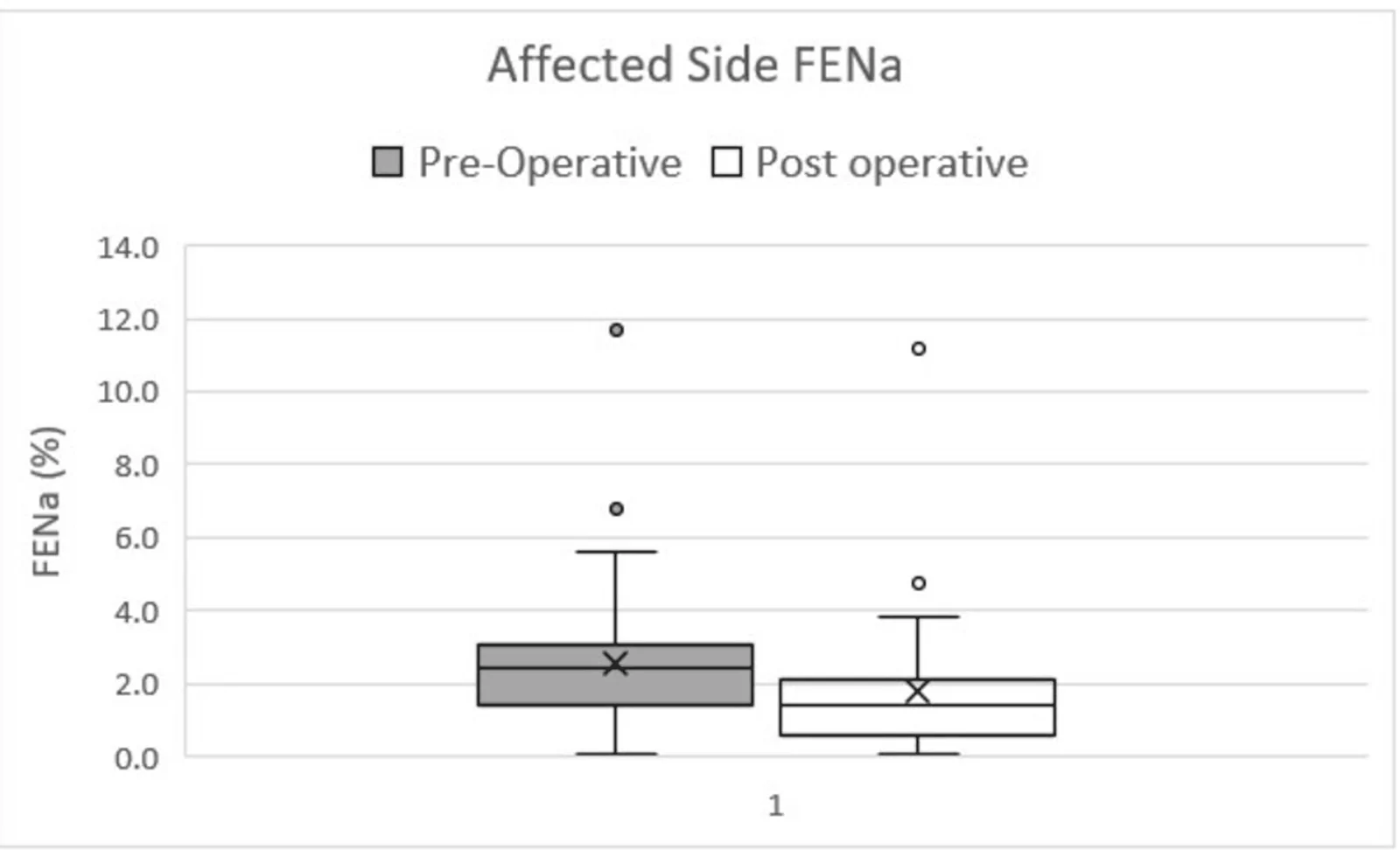
Box-and-whisker plot showing the fractional excretion of sodium (FENa) of the affected kidney before (gray-shaded box) and after (white box) pyeloplasty. The horizontal line within each box denotes the median, the “X” mark represents the mean, and circles indicate outliers.

The FENa on the affected side decreased significantly following surgery, from a preoperative value of 2.563 ± 2.146% to a postoperative value of 1.809 ± 1.977% (p < 0.001) (Figure 3). In our study, most children with a preoperative FENa of <3% demonstrated greater postoperative improvement than those with a preoperative FENa of >3%; however, this difference did not reach statistical significance. 

Correlation between FENa and DRF on renal scintigraphy

**Figure 4:**
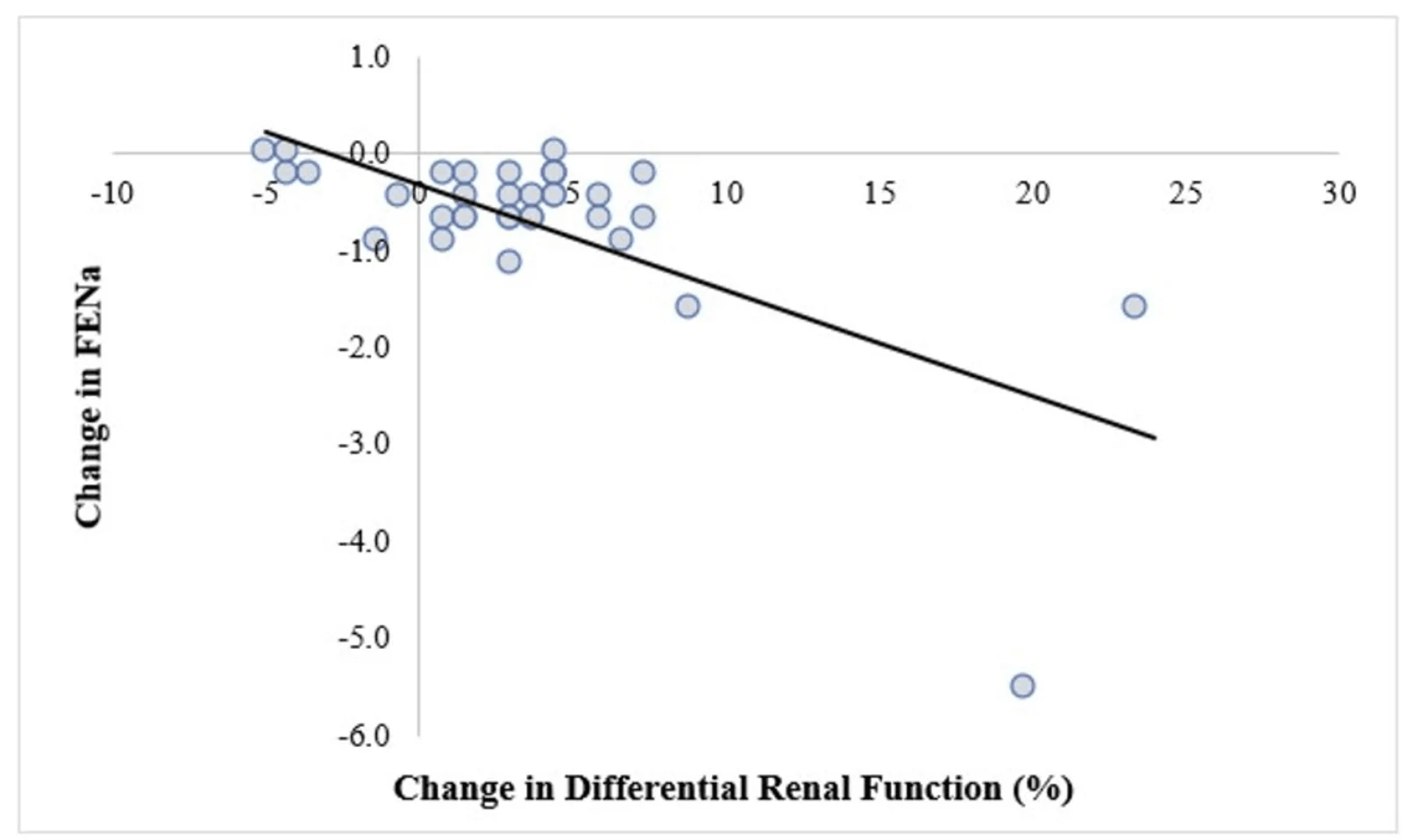
Scatter plot showing the correlation between change in fractional excretion of sodium (FENa) and change in differential renal function (DRF) of the affected kidney after pyeloplasty.

The change in FENa between the preoperative and postoperative assessments was calculated and correlated with the corresponding change in DRF, expressed as a percentage. The change for each parameter was defined as the postoperative value minus the preoperative value. A significant negative correlation (r = -0.650) indicated that a greater postoperative reduction in FENa was associated with a greater improvement in DRF (p < 0.001) (Figure 4).

Correlation between FENa and APPD on renal USG

**Figure 5:**
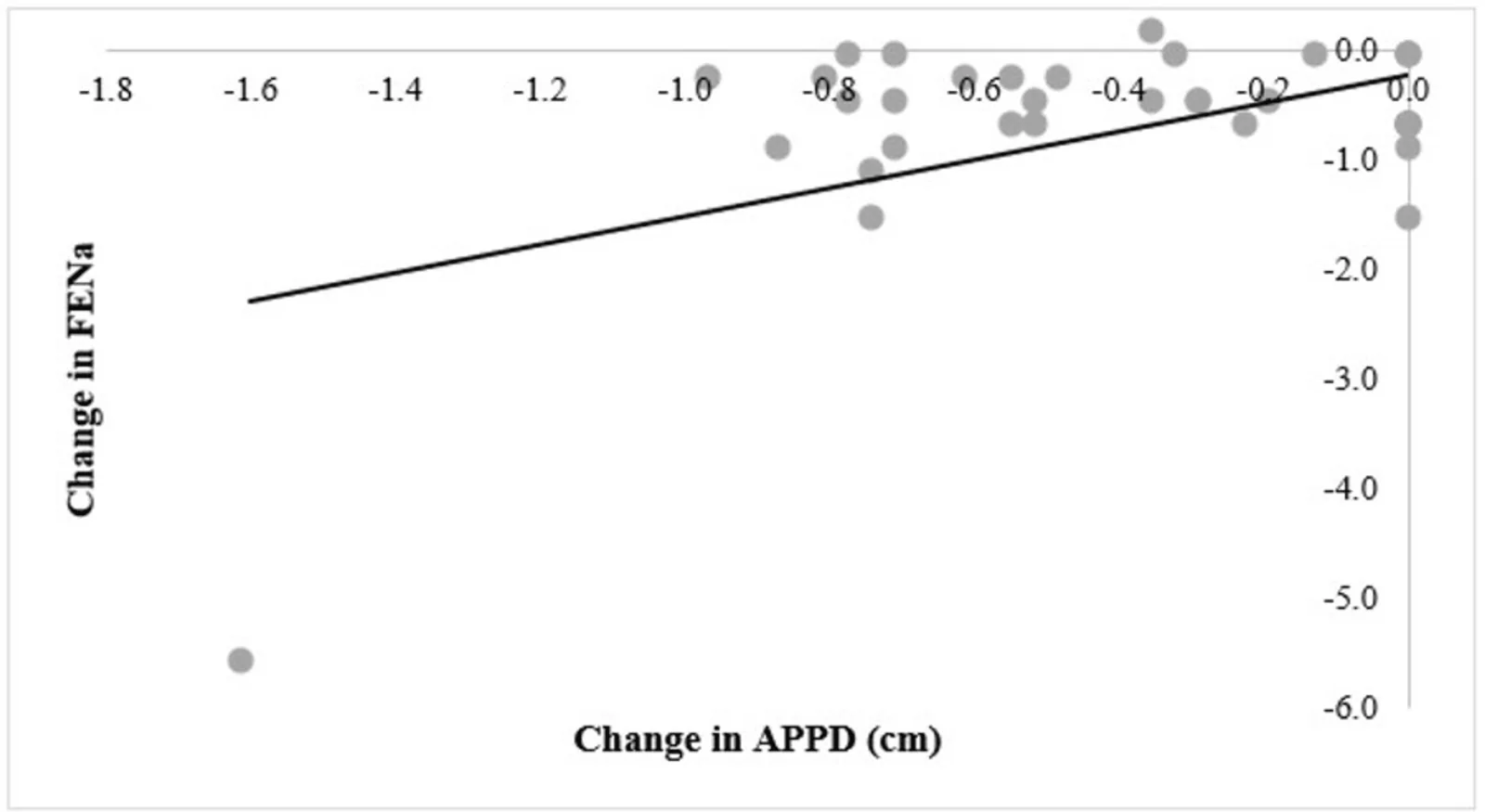
Scatter plot showing the correlation between change in fractional excretion of sodium (FENa) and change in anteroposterior pelvic diameter (APPD) of the affected kidney in ultrasonography after pyeloplasty.

The change in FENa between the preoperative and postoperative assessments was calculated and correlated with the corresponding change in APPD, expressed in centimeters. The change for each parameter was defined as the postoperative value minus the preoperative value. A significant positive correlation (r = 0.506) indicated that a greater postoperative reduction in FENa was associated with a greater reduction in APPD (p = 0.001) (Figure 5).

Correlation between FENa and cortical thickness on renal USG

**Figure 6:**
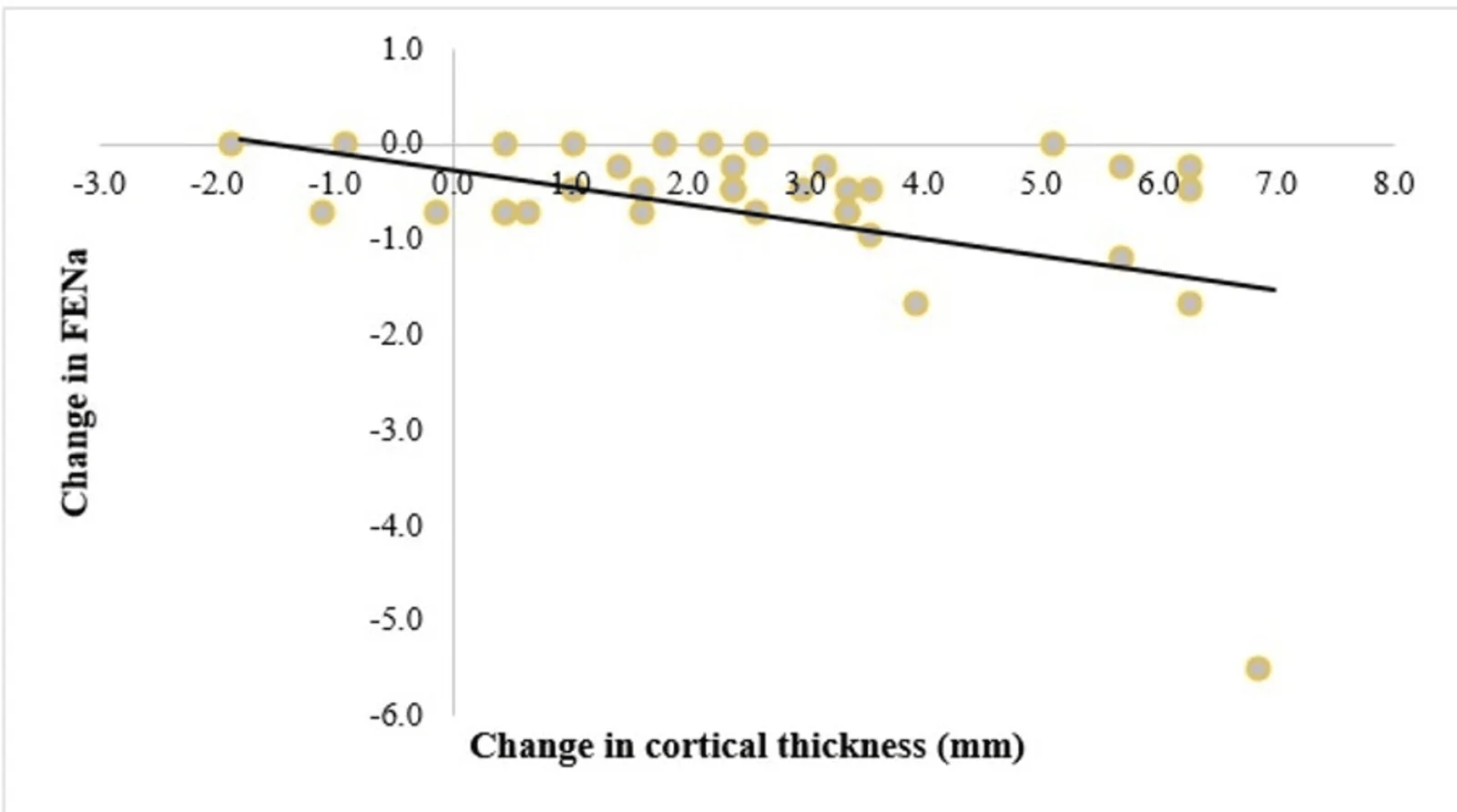
Scatter plot showing the correlation between change in fractional excretion of sodium (FENa) and change in cortical thickness of the affected kidney in ultrasonography after pyeloplasty.

The change in FENa between the preoperative and postoperative assessments was calculated and correlated with the corresponding change in renal cortical thickness, expressed in millimeters. The change for each parameter was defined as the postoperative value minus the preoperative value. A significant negative correlation (r = -0.413) indicated that a greater postoperative reduction in FENa was associated with a greater increase in renal cortical thickness (p = 0.009) (Figure 6).

## Discussion

UPJO is recognized as one of the leading causes of hydronephrosis in the pediatric population. In the absence of timely intervention, it may result in progressive and irreversible deterioration of renal function in the affected kidney [1]. USG remains the initial imaging modality of choice, as it provides valuable information regarding calyceal configuration, the APPD, and renal cortical thickness [1]. Radionuclide renography is a key investigation for assessing the differential renal function of each kidney and identifying urinary tract obstruction. It is widely regarded as the gold standard for evaluating the severity of UPJO [1]. Radionuclide renography provides both qualitative and quantitative parameters for the diagnosis of UPJO [6-9]. These parameters include T½, DRF, gravity-assisted drainage, and postvoid images, all of which are also useful for evaluating postoperative outcomes [10-14].

The FENa has been extensively studied in adults as a marker of AKI. It is traditionally used to differentiate the underlying etiology of AKI. In prerenal AKI caused by volume depletion, sodium reabsorption is maximally stimulated, resulting in a characteristically low FENa, whereas an FENa > 1% is generally suggestive of tubular dysfunction [5,15].

In the present study, DRF of the affected kidney improved significantly after surgery, increasing from 39.63 ± 8.63% preoperatively to 43.60 ± 5.82% postoperatively (p < 0.001). Six patients demonstrated a postoperative decline in DRF of ≤5% compared with their preoperative values. However, all of these patients had a normal postoperative drainage pattern, normal T½, remained asymptomatic, and demonstrated improved renal cortical thickness. None required redo pyeloplasty. Furthermore, both T½ and Tmax of the affected kidney decreased significantly following surgery (p < 0.001), indicating improved urinary drainage, consistent with previous reports [9,14,16].

Chiou et al. prospectively evaluated 38 children with unilateral UPJO and compared several urinary biomarkers, including the fractional excretion of sodium, potassium, calcium, phosphorus, magnesium, tumor necrosis factor-α (TNF-α), transforming growth factor-β1 (TGF-β1), epidermal growth factor (EGF), and fragmented deoxyribonucleic acid (DNA) as a marker of apoptosis [4]. Patients were stratified according to whether postoperative DRF was <40% or ≥40%. Samples from the affected kidney were obtained through PCN, whereas bladder urine was used to represent the contralateral kidney. Fractional excretion was calculated as (urinary solute concentration/serum solute concentration) × 100. Among the biomarkers evaluated, only the fractional excretion of sodium and chloride correlated with postoperative DTPA findings. The mean fractional excretion of sodium was 1.77 ± 0.30% in the affected kidney and 1.15 ± 0.52% in the contralateral kidney. Values were 1.16 ± 0.22% in children with preserved renal function (postoperative DRF ≥ 40%) and 2.66 ± 0.70% in those with impaired renal function (postoperative DRF <40%). The authors concluded that the fractional excretion of sodium in urine obtained from a hydronephrotic kidney may serve as a convenient marker for screening renal injury and confirming obstruction. However, only preoperative samples were analyzed in their study [4].

In the present study, FENa of the affected kidney decreased significantly after pyeloplasty, from 2.563 ± 2.146% preoperatively to 1.809 ± 1.977% postoperatively (p < 0.001). Bladder urine collected through urethral catheterization before surgery was used as a surrogate for the contralateral, unaffected kidney. The FENa of the unaffected side also decreased significantly, from 2.040 ± 0.505% preoperatively to 1.434 ± 0.696% postoperatively (p < 0.001). In addition, urinary sodium concentration from the affected kidney decreased significantly after surgery, from 87.51 ± 30.19 to 79.03 ± 35.66 (p = 0.013).

Two adolescents (aged 15 and 16 years) presented with severely impaired renal function (baseline DRF ≤ 10%) on 99mTc-DTPA renography, precluding assessment of drainage because of poor tracer uptake. These patients underwent PCN insertion. Serial urinary sodium measurements were obtained from the nephrostomy tube at approximately two-month intervals and were compared with repeat DTPA findings. Both patients demonstrated marked improvement in renal function (DRF increased to 30% and 34%, respectively) and subsequently underwent pyeloplasty. Correspondingly, FENa decreased from 6.8% and 3.2% preoperatively to 1.1% and 1.5% postoperatively, paralleling the improvement in DRF.

Schreuder et al. evaluated 761 paired urine and serum samples from children attending their clinic to investigate sodium excretion, creatinine clearance, and FENa [15]. Among these, only five patients had generalized tubular dysfunction, allowing assessment of the relationship between tubular function and FENa. The authors demonstrated that FENa is influenced by both glomerular filtration rate and sodium intake and concluded that interpretation of FENa requires adjustment for these factors. Consequently, they were unable to establish a definitive cutoff value for abnormal tubular sodium handling [15].

In the present study, a significant negative correlation was observed between changes in FENa and DRF (r = -0.650, p < 0.001), indicating that greater reductions in FENa were associated with greater improvements in DRF. A significant positive correlation was found between changes in FENa and APPD (r = 0.506, p = 0.001), demonstrating that greater reductions in FENa were associated with greater reductions in APPD. Likewise, a significant negative correlation was observed between changes in FENa and renal cortical thickness (r = -0.413, p = 0.009), indicating that greater reductions in FENa were associated with greater increases in cortical thickness. In contrast, no significant correlations were identified between FENa and T½ (p = 0.231), drainage pattern (p = 0.807), or grade of hydronephrosis (p = 0.232).

Several urinary biomarkers, including neutrophil gelatinase-associated lipocalin (NGAL), monocyte chemoattractant protein-1 (MCP-1), carbohydrate antigen 19-9 (CA 19-9), kidney injury molecule-1 (KIM-1), epidermal growth factor (EGF), and interferon gamma-induced protein-10 (IP-10), have been investigated to distinguish obstructive UPJO from non-obstructive hydronephrosis. Among these, the combination of NGAL, KIM-1, and CA 19-9 has demonstrated the most promising diagnostic performance. However, their routine clinical application remains limited by high cost, methodological heterogeneity, relatively small study populations, and the absence of standardized cutoff values, restricting their use primarily to research settings [17].

Collectively, these findings suggest that the fractional excretion of sodium may serve as a useful adjunctive marker for assessing renal function in children with UPJO and for monitoring postoperative functional recovery following pyeloplasty.

This study has several limitations. First, it was conducted at a single tertiary care center with a relatively small sample size of 35 patients. Although the sample size was determined by the study period, an a priori sample size calculation with power analysis would have strengthened the statistical robustness of the findings. Second, the bladder urine sample used to represent the contralateral kidney did not exclusively originate from the unaffected kidney, precluding precise determination of normal-side FENa values. Third, collection of postoperative urine samples during DJ stent removal required bilateral ureteric catheterization, which increased procedural complexity and operative time. Fourth, we were unable to determine a statistically significant FENa cutoff value for predicting postoperative renal functional recovery. Finally, the relatively short follow-up period precluded assessment of long-term renal functional outcomes after pyeloplasty.

To our knowledge, this is the first prospective study to evaluate the relationship between fractional excretion of sodium and renal ultrasonographic and scintigraphic parameters before and after pyeloplasty in children with UPJO.

## Conclusions

FENa provides an integrated assessment of renal function by reflecting both glomerular function (through creatinine clearance) and tubular function (through sodium reabsorption and excretion). It may serve as a surrogate marker of preoperative renal impairment in patients with unilateral UPJO. A significant correlation was observed between FENa and DRF both before and after pyeloplasty; however, no significant correlation was demonstrated with the drainage pattern. A practical limitation of FENa is that urine samples representative of the affected kidney can only be obtained intraoperatively, thereby limiting its utility as a preoperative prognostic marker.
